# Incorrect use of inhalation devices among patients with bronchial asthma. A hospital-based cross-sectional study in Rio de Janeiro, Brazil

**DOI:** 10.1590/1516-3180.2018.0050170418

**Published:** 2018-08-13

**Authors:** Carlos Leonardo Carvalho Pessôa, Maria Julia da Silva Mattos, Artur Renato Moura Alho, Marianna Martini Fischmann, Ana Carolina Castro Côrtes, Flávio de Oliveira Mendes, Bruno Mendes Haerdy, Sandra Mara Silva Brignol

**Affiliations:** I MD, MSc, PhD. Adjunct Professor, Department of Clinical Medicine, Universidade Federal Fluminense (UFF), Niterói (RJ), Brazil.; II Medical Student, Universidade Federal Fluminense (UFF), Niterói (RJ), Brazil.; III Medical Student, Universidade Federal Fluminense (UFF), Niterói (RJ), Brazil.; IV Medical Student, Universidade Federal Fluminense (UFF), Niterói (RJ), Brazil.; V Medical Student, Universidade Federal Fluminense (UFF), Niterói (RJ), Brazil.; VI Medical Student, Universidade Federal Fluminense (UFF), Niterói (RJ), Brazil.; VII Medical Student, Universidade Federal Fluminense (UFF), Niterói (RJ), Brazil.; VIII BSc, MSc, PhD. Adjunct Professor, Department of Epidemiology and Biostatistics, Institute of Public Health, Universidade Federal Fluminense (UFF), Niterói (RJ), Brazil.

**Keywords:** Asthma, Metereddose inhalers, Dry powder inhalers

## Abstract

**CONTEXT AND OBJECTIVE:**

Treatment of asthma implies inhalation of specific drugs to reach high concentrations in the respiratory tree and ensure low drug bioavailability and few adverse effects. This study aimed to evaluate the effectiveness of the inhalation technique among outpatients with asthma.

**DESIGN AND SETTING:**

Tertiary-care hospital-based cross-sectional study in Rio de Janeiro.

**METHODS:**

We evaluated inhalation practices in a convenience sample. A questionnaire was used to investigate sociodemographic data and assess disease control level, duration of use of the inhalation device, length of treatment and previous instructions provided by the prescribing physician. Patients demonstrated their inhalation technique using empty devices, and their technique was considered correct when all steps were appropriately performed or when errors did not interfere with the treatment outcome.

**RESULTS:**

Among the 71 participants, 53 (74.7%) had been using the same inhaler device for at least two years and 41 (57.8%) had been under treatment for two years or more. Twelve (17.1%) said that they had been taught once and 57 (81.4%) at least twice, while one (1.4%) reported not having received any guidance regarding use of inhaler devices. Eighteen patients (25.3%) presented controlled asthma and 28 (39.5%) performed the inhalation technique correctly. Incorrect technique was associated with fewer evaluations of the inhalation technique (P =0.04) and uncontrolled asthma (P = 0.01).

**CONCLUSIONS:**

Less than half of the sample performed the inhalation technique correctly. Incorrect inhalation technique was related to lower number of evaluations of the use of the inhalation device and uncontrolled asthma.

## INTRODUCTION

Asthma affects up to 300 million people worldwide^1^ and, in 2015, caused one to two deaths per day in Brazil.^2^

Use of inhaler therapy allows drugs to rapidly reach high concentrations in the airways, with therapeutic effects even with low drug bioavailability and few adverse effects. Inhaler devices are therefore the main route of administration for treatments for asthma.^3^^,^^4^ The goals of asthma treatment are symptom control and future risk reduction.^5^^,^^6^ However, improper use of an inhaler device can substantially reduce treatment effectiveness, thus resulting in uncontrolled disease, side effects, higher costs,^7^^,^^8^ greater need for rescue medications, more emergency visits and hospitalizations, and low adherence to treatment.^9^^,^^10^ Previous studies have shown an association between disease control and proper use of inhaler devices.^11^^,^^12^

The guidelines recommend regular monitoring of the inhalation technique.^6^^,^^13^ Despite this, sometimes only 25% of patients receive verbal and visual guidance regarding the inhalation technique, and when guidance occurs, it is often of poor quality.^14^^,^^15^ Lack of time, use of poorly comprehensible language and neglect of reassessment interfere with the inhalation technique instructions given by healthcare professionals, and these factors increase the risk of misunderstandings.^13^

This study aimed to evaluate the effectiveness of the inhalation technique among outpatients with asthma.

## METHODS

### Study design, setting and ethics

This was a cross-sectional observational study on a convenience sample of patients with bronchial asthma who were being treated at one of the outpatient clinics of the Antônio Pedro University Hospital (Hospital Universitário Antônio Pedro, HUAP) in Niterói, state of Rio de Janeiro, Brazil. This study was approved by the local ethics committee of our institution (Universidade Federal Fluminense, UFF), under the number CAAE 56248816.1.0000.5243. Patients signed informed consent forms for their participation in this study.

### Participants

Patients were eligible if they were 18 years of age or older, were not at their first medical appointment and were using the following inhaler devices containing any corticoids, long-acting bronchodilators (or combinations of them) or even a short-acting bronchodilator: Aerolizer, Aerocaps, Diskus and metered-dose inhaler (MDI), without the aid of a spacer. Patients in a phase of exacerbation of symptoms were excluded and referred to the emergency room for immediate treatment. Patients whose diagnoses of asthma were not confirmed during the treatment and those who regularly received any help from others for using the inhaler device were also excluded.

Recruitment was performed sequentially, according to the scheduling dates of patients’ medical appointments. After signing an informed consent form, patients gave responses to a self-administered questionnaire that sought sociodemographic data. They had to demonstrate to researchers how they used the device.

Patients’ degree of disease control was evaluated in accordance with the Global Initiative for Asthma (GINA)^6^ document, and their length of time of use of the inhaler device, their treatment duration, the existence of instructions regarding use of the device issued by the prescribing physician and the existence of regular supervision of the inhalation technique at subsequent visits were assessed. Spirometry was performed and assessed in accordance with the Brazilian Guidelines for Pulmonary Function Tests.^16^

In our outpatient clinic, patients are given guidance for the inhalation technique, reinforced at all visits. The physician always demonstrates the technique using an empty device equivalent to the one used by the patient. It is always requested that patients should bring their empty devices for training, and when they do, they can practice the inhalation technique in front of the physician, and if there are any errors, these are always corrected.

The inhalation technique was assessed according to the following check-list: for Diskus users, the user was required to open the inhaler, push the lever back completely, exhale to residual volume, inhale deeply, hold breath for 10 seconds, exhale away from mouthpiece and close the inhaler. For MDI users, it was necessary to remove the mouthpiece cover, hold the inhaler upright, exhale to residual volume, press the canister and inhale deeply, hold breath for 10 seconds, exhale away from the mouthpiece and close the inhaler. For Aerolizer or Aerocaps users, it was necessary to remove the inhaler cover, open the capsule compartment, place the capsule in the appropriate chamber, close the capsule compartment, press the button(s) of the inhaler, exhale to residual volume, inhale deeply, hold breath for 10 seconds, exhale away from the mouthpiece and close the inhaler.

### Evaluations

Only the inhalation technique that was performed using the device that patients considered to be their main device was evaluated in this study. The patients demonstrated their technique using empty devices, to at least two evaluators (one pulmonologist and one of a group of six medical students who had been trained by the pulmonologist). The inhalation technique demonstrations were filmed for reevaluation, for the eventuality of any disagreement among the evaluators. In such cases, evaluation by a third evaluator was mandatory.

The evaluation of the inhalation technique was based on the leaflet provided by the producer of each device. The technique was considered correct when all steps were performed properly or when the misconceptions probably did not interfere with the outcome of the treatment (e.g. not closing the device at the end of the demonstration). The technique was considered incorrect when one or more errors were observed in any of the following steps: preparation of the inhalation device for use, expiration, aspiration or apnea, regardless of the severity of the error. Whenever there were misconceptions relating to the inhalation technique, the patient received instructions on the correct use of the inhalation device.

### Statistical analysis

The data obtained were digitized in a Microsoft Excel 2010 worksheet and were exported, for statistical analysis, into the Epi Info 7.2 data analysis software. Central trend measurements (average and median) and dispersion measurements were used for descriptive analysis on the numerical data. Simple and relative frequency calculations were used for questions with categorical variables. The chi-square test or Fisher’s exact test was used to verify associations between the quality of the inhalation technique and patient characteristics, asthma control, severity of obstruction, number of evaluations of inhalation technique that had been made and duration of treatment. P-values (descriptive levels) lower than 0.05 were taken to indicate significant associations. A binary logistic regression model was used to identify characteristics that were predictive of incorrect technique. Variables with a significance level of less than 0.1 in univariate analysis, after adjustment for gender and age, were included in a logistic regression model.

## RESULTS

Between August 2, 2016, and March 10, 2017, 75 patients were enrolled. However, four were excluded: one had an immobilized arm, which prevented unaided demonstration of the inhalation technique; one was subsequently confirmed to have a diagnosis of chronic obstructive pulmonary disease (instead of asthma); and two used the Aerocaps device when they should have been using Aerolizer to administer their medications (due to modification of the medications during the study, but using the “old” inhaler, inappropriately).

The final sample was thus composed of 71 participants between 19 and 81 years old, with a mean age of 57.7 ± 13.9 years, of whom 61 (85.9%) were female. Fourteen (19.7%) were single, 31 (43.7%) were married and 26 (36.6%) were separated, divorced or widowed. Thirty-seven (52.1%) had at most completed elementary education (9 years of schooling), 29 (40.8%) had at most obtained a high school diploma and five (7.1%) had obtained a university degree at bachelor or higher level. Regarding personal income, 49 (70%) received a maximum of one Brazilian minimum salary per month (equivalent to approximately US$ 247,46), whereas 20 (28.6%) received between one and three minimum salaries and one (1.4%) earned between three and ten minimum salaries. Regarding family income, 28 (40%) received up to one minimum salary, 38 (54.3%) one to three minimum salaries and four (5.7%) between three and ten minimum salaries.

According to the GINA document, two participants (2.8%) were in stage 1 of the treatment of bronchial asthma (as needed short-acting beta2-agonist), 14 (19.7%) in stage 2 (low dose of inhaled corticosteroids), 11 (15.5%) in stage 3 (low dose of inhaled corticosteroids and long-acting bronchodilator), 43 (60.6%) in stage 4 (moderate or high dose of inhaled corticosteroids and long-acting bronchodilator) and one (1.4%) in stage 5 (oral corticosteroids and/or anti IgE associated to Inhaled medications used in Stage 4). With regard to the asthma control level, only 18 patients (25.35%) presented controlled asthma, while 20 (28.15%) exhibited partial control over the disease and 33 (46.5%) had uncontrolled asthma. Concerning the severity of the obstructive disorder, 50 (70.42%) had spirometry findings that were considered normal or presented mild obstruction, 13 (18.31%) presented moderate disorder and six (8.45%) had severe obstruction. Two patients (2.82%) were not able to perform the test reliably. Fifty-six patients (78.9%) reported using the prescribed medication in accordance with the physician’s recommendation and nine (12.7%) only rarely failed to use the drugs.

Regarding the inhaler devices, 10 patients (14.1%) used Aerolizer, 36 (50.7%) Aerocaps, 6 (8.5%) Diskus and 19 (26.7%) MDI. Fifty-three patients (74.7%) had been using the same device for at least two years and forty-one (57.8%) had been undergoing treatment at the outpatient clinic for two years or more, always coordinated by the same pulmonology specialist.

Only one participant (1.4%) reported not receiving any guidance at any time regarding use of the inhalation device, while 13 (18.3%) were never reassessed after their first instruction, 25 (80.3%) were reassessed once at an appointment subsequent to their first instruction and 32 (45.1%) were reassessed at least twice at medical appointments subsequent to their first instruction. This last group was therefore instructed at least three times.

Concerning the quality of the inhalation technique, 28 (39.5%) performed it correctly. In the few cases in which there was initial disagreement among the evaluators, the video review always unified opinions about the assessment of the inhalation technique. Incorrect inhalation technique showed associations with the asthma control level (P = 0.01) and the number of instructions (P = 0.04). However, there were no associations between incorrect inhalation technique and gender, age, marital status, personal and family income, level of education, duration of treatment orseverity of obstructive disturbance (Table 1). After controlling for all other factors, as described in the methods section, only the risk factors of uncontrolled asthma and fewer instructions remained associated with inadequate use of the device.

**Table 1:** Factors relating to the quality of the inhalation technique

Variable	Inhalation technique		
	Incorrect (n/%)	Correct (n/%)	P- value
Asthma control level			
Controlled/partially controlled	18 (25.3)	20 (28.2)	0.01*
Uncontrolled	25 (35.2)	8 (11.3)	
Gender			
Male	6 (8.5)	4 (5.6)	0.61
Female	37 (52.1)	24 (33.8)	
Age (years)			
< 50	14 (19.7)	5 (7.0)	0.17
≥ 50	29 (40.9)	23 (32.4)	
Marital Status			
Married	17 (24.0)	14 (19.7)	0.38
Single/divorced/separated/widowed	26 (36.6)	14 (19.7)	
Educational level			
Elementary education at most	26 (36.6)	11 (15.5)	0.08
Incomplete high school at least	17 (23.95)	17 (23.95)	
Personal income			
One minimum salary/month at most	32 (45.7)	17 (24.3)	0.16
More than one minimum salary/month	10 (14.3)	11 (15.7)	
Family income			
One minimum salary/month at most	19 (27.15)	9 (12.85)	0.27
More than one minimum salary/month	23(32.9)	19 (27.1)	
Severity of obstructive disorder			
Mild	28 (40.6)	22 (31.9)	0.34
Moderate or severe	13 (18.8)	6 (8.7)	
Years of use of inhalation device			
≥ 2	29 (40.9)	24 (33.8)	0.07
< 2	14 (19.7)	4 (5.6)	
Number of orientations			
≥ 2	31 (44.3)	26 (37.1)	0.04*
≤ 1	11 (15.7)	2 (2.9)	

*Significance level P < 0.05.

## DISCUSSION

Lack of information is the main cause of improper use of inhalers,^17^ and a simple statement given by a patient that he performs the inhalation technique properly, without any demonstration, is not necessarily a guarantee of good performance.^14^ Reports of absence of instruction and reassessment of the inhalation technique are not uncommon. In at least two studies,^14^^,^^18^ only approximately 66% of the patients were initially taught; in another study,^19^ 90% were instructed at the first visit, but only 14% were reassessed on other occasions. In our study, almost all patients reported having been given guidance at least once and approximately 80% had been trained at least twice, but only 40% of the patients performed the inhalation technique properly. This result is similar to what has already been published from some studies,^17^^,^^20^^,^^21^ but the percentage reported here is greater than what has been reported in several other studies, in which only 6% to 31% of the participants performed it correctly.^13^^,^^15^^,^^22^^,^^23^^,^^24^

Unlike several previous studies,^11^^,^^18^^,^^25^^,^^26^ no relationship was found in our study between old age and incorrect inhalation technique. This was also reported in one other study.^22^ The continued practice that is provided at our outpatient center probably helps to explain this outcome, because training among elderly patients also results in significant improvement in the inhalation technique.^8^ Previously, a relationship between inadequate inhalation technique and widowhood^22^ was also demonstrated, but this was not confirmed in our current study. There is evidence for associations between incorrect technique and low educational and income levels.^9^^,^^18^^,^^22^ However, we did not observe these associations. We could not confirm the relationship between obstructive disorders of greater severity and incorrect inhalation technique that has previously been noted.^25^ Patients with severe and moderate obstructions made errors more frequently than did those with mild obstruction or normal results in the spirometry test, but this difference was not statistically significant. Errors in inhalation technique were frequent, regardless of the socioeconomic, educational or obstruction level, which emphasizes the need to supervise the quality of the technique for any patient profile.

The length of time for which the treatment had been used did not interfere with the quality of the inhalation technique, as already reported.^27^ However, there was an effect from the number of evaluations performed. This observation was supported by previous studies,^17^^,^^26^ which showed that patients who had never been given any guidance frequently made errors in the inhalation technique. Furthermore, a single explanation of the technique may be insufficient. In such cases, at most 48.4% performed the technique correctly, but only if they had received extensive orientation at the first visit.^28^ However, they reached their maximum ability if three instruction sessions were provided, such that the error rate might be less than 10%.^20^ In the present study population, only 45.6% of the patients who were given guidance twice or more showed correct technique. Takaku et al.^20^ worked with patients who received a prescription of an inhaled drug for the first time, for whom the technique was taught and supervised successively for between two and five times at intervals of two weeks to one month. This was a shorter period with more intensive training than in our study. Nevertheless, both studies emphasize the need for instructions given on multiple occasions, in order to achieve the proper inhalation technique. It is possible that instruction repetition is the main key to achieving asthma control.

Regarding the limitations of our study, besides its cross-sectional design and the sample size, the presence of comorbidities was not considered in this investigation. The evaluations were not performed under blinded conditions, and the investigators always analyzed the quality of the inhalation technique together. Adherence to treatment was investigated only through basic questions, and non-adherence might be a confounding factor that, in addition to inadequate technique, may have contributed to the causes of uncontrolled asthma. However, if it is assumed that the patients answered honestly regarding their adherence to treatment, more than 90% were performing the treatment properly. This is an excellent result and therefore would not be a reason for asthma control not to be obtained. The logistic regression model used did not change the results previously obtained, perhaps because of the sample size. Finally, our study was based on a convenience sample in a hospital that is equipped to deal with cases of high complexity, i.e. cases that are more difficult to control are commonly treated there. Thus, the results from this study may not represent the situations for other hospitals, and the ability to generalize from these results is limited.

This study did not show any association between the inhalation technique and any of the socioeconomic characteristics, the severity of obstruction, or even the duration of the treatment. One of the only relationships observed was between inadequate technique and uncontrolled asthma. Patients who received a greater number of orientations were also observed to be better at the inhalation technique. There seems to be a single simple conclusion: if patients adhere to the treatment using the correct inhalation technique, their asthma will be controlled. For this to occur, caregivers must insist on teaching the technique regardless of the patient’s profile. This seems to be the only way to achieve the main goal of treating asthma, namely, achievement of control.

## CONCLUSIONS

The inhalation technique was correctly performed by 39.5% of the participants. Uncontrolled asthma was significantly associated with incorrect inhalation technique. The incorrect inhalation technique was observed frequently and had no relationship with age, gender, marital status, educational level, personal and family income, duration of use of the inhalation device or the severity of obstruction. This study emphasizes the need for quality supervision of the inhalation technique and suggests that the larger the number of reevaluations is, the closer it will be possible to come to proper execution of the technique and asthma control. Inhalation technique should be supervised for all patients, especially those who received fewer instructions and those with uncontrolled asthma.

## References

[1] World Health Organization (2007). Global surveillance, prevention and control of chronic respiratory diseases: a comprehensive approach.

[2] Brasil. Ministério da Saúde (2016). Sistema de Informações Hospitalares do SUS (SIH/SUS).

[3] Virchow JC, Crompton GK, Dal Negro R (2008). Importance of inhaler devices in the management of airway disease. Respir Med.

[4] Lötvall J (2001). Inhalation therapy of the future--how will it change the way we treat asthma?. J Aerosol Med.

[5] Przybyszowski M, Bochenek G (2015). The role of questionnaires in the assessment of asthma control. Pneumonol Alergol Pol.

[6] Global Initiative for Asthma (2014). Global strategy for asthma management and prevention (c) 2014. Glob Initiat Asthma.

[7] Basheti IA, Qunaibi E, Bosnic-Anticevich SZ (2011). User Error with Diskus and Turbuhaler by Asthma Patients and Pharmacists in Jordan and Australia. Respir Care.

[8] Crane MA, Jenkins CR, Goeman DP, Douglass JA (2014). Inhaler device technique can be improved in older adults through tailored education: findings from a randomised controlled trial. NPJ Prim Care Respir Med.

[9] Melani AS, Bonavia M, Cilenti V (2011). Inhaler mishandling remains common in real life and is associated with reduced disease control. Respir Med.

[10] Melani AS, Zanchetta D, Barbato N (2004). Inhalation technique and variables associated with misuse of conventional metered-dose inhalers and newer dry powder inhalers in experienced adults. Ann Allergy, Asthma Immunol.

[11] Coelho AC, Souza-Machado A, Leite M (2011). Manuseio de dispositivos inalatórios e controle da asma em asmáticos graves em um centro de referência em Salvador [Use of inhaler devices and asthma control in severe asthma patients at a referral center in the city of Salvador, Brazil]. J Bras Pneumol.

[12] Haughney J, Price D, Kaplan A (2008). Achieving asthma control in practice Understanding the reasons for poor control. Respir Med.

[13] Manríquez P, Acuña AM, Muñoz L, Reyes A (2015). Study of inhaler technique in asthma patients: differences between pediatric and adult patients. J Bras Pneumol.

[14] Souza MLDM, Meneghini AC, Ferraz E, Vianna EO, Borges MC (2009). Técnica e compreensão do uso dos dispositivos inalatórios em pacientes com asma ou DPOC [Knowledge of and technique for using inhalation devices among asthma patients and COPD patients]. J Bras Pneumol.

[15] Ganguly A, Das AK, Roy A (2014). Study of Proper use of Inhalational Devices by Bronchial Asthma or COPD Patients Attending a Tertiary Care Hospital. J Clin Diagn Res.

[16] Pereira CA de C (2002). Diretrizes para Testes de Função Pulmonar. J Bras Pneumol.

[17] Al-Jahdali HH, Ahmed A, Al-Harbi A (2013). Improper inhaler technique is associated with poor asthma control and frequent emergency department visits. Allergy Asthma Clin Immunol.

[18] Oliveira PD, Menezes AM, Bertoldi AD, Wehrmeister FC, Macedo SE (2014). Assessment of inhaler techniques employed by patients with respiratory diseases in southern Brazil: a population-based study. J Bras Pneumol.

[19] Basheti IA, Obeidat NM, Ammari WG, Reddel HK (2016). Associations between inhaler technique and asthma control among asthma patients using pressurised MDIs and DPIs. Int J Tuberc Lung Dis.

[20] Takaku Y, Kurashima K, Ohta C (2017). How many instructions are required to correct inhalation errors in patients with asthma and chronic obstructive pulmonary disease?. Respir Med.

[21] Bettencourt ARDC, Oliveira MA, Fernandes ALG, Bogossian M (2002). Educação de pacientes com asma: atuação do enfermeiro [Education of the asthmatic patient: the nursing approach]. J Pneumol.

[22] DalcinPde T, Grutcki DM, Laporte PP (2014). Fatores relacionados ao uso incorreto dos dispositivos inalatórios em pacientes asmáticos [Factors related to the incorrect use of inhalers by asthma patients]. J Bras Pneumol.

[23] Sanchis J, Gich I, Pedersen S, Aerosol Drug Management Improvement Team (ADMIT) (2016). Systematic Review of Errors in Inhaler Use: Has Patient Technique Improved over Time?. Chest.

[24] Molimard M, Raherison C, Lignot S (2003). Assessment of handling of inhaler devices in real life: an observational study in 3811 patients in primary care. J Aerosol Med.

[25] Wieshammer S, Dreyhaupt J (2008). Dry powder inhalers: which factors determine the frequency of handling errors?. Respiration.

[26] Maricoto T, Madanelo S, Rodrigues L (2016). Educational interventions to improve inhaler techniques and their impact on asthma and COPD control: a pilot effectiveness-implementation trial. J Bras Pneumol.

[27] Verver S, Poelman M, Bögels A, Chisholm SL, Dekker FW (1996). Effects of instruction by practice assistants on inhaler technique and respiratory symptoms of patients. A controlled randomized videotaped intervention study. Fam Pract.

[28] Sandrini A, Jacomossi A, Farensin SM, Fernandes ALG, Jardim JR (2001). Aprendizado do uso do inalador dosimetrado após explicação por pneumologista. J Pneumol.

